# Medicine vs. Advice

**Published:** 1871-01

**Authors:** 


### MEDICINE VS. ADVICE.

It would seem that when a physician is summoned to a sick chamber, that both the invalid and the family expect the administration of some nauseous dose of medicine, else are not the requirements of the visitation complete. The truth is, there exists a great demand for medicines among all classes of community, for diseases either fancied or real; and the physician who seeks to cure his patient by good and evidently required, hygienic advice, falls far short of the mark, if he expects to acquire the reputation of a popular medical man.

Should the physician be called to attend a sufferer from a fit of indigestion—one who is terribly annoyed by an irregular beating of the heart, and, with finger upon his pulse, is counting every intermission it makes until the doctor arrives,—what folly it would be to direct *that* man to eat less, to eat one or two meals a day, and those to consist of graham bread, boiled wheat, and milk, or a poached egg with toast, until he had sufficiently rested his over burdened stomach to enable it to resume its labors in a becoming manner!

Why, that chap wants *medicine*. You can see it in his eye, and in the expression of disappointment and perhaps disgust, which you may read in his face while you are telling him how to cure his difficulty effectually, and how to prevent its recurrence. Leave him without medicine, and you leave him forever, for he will be sure to summon some doctor willing and anxious to prescribe the much coveted, medical draught.

A physician's advice amounts to nought unless coupled with a dose of medicine. Then, most persons are more willing to pay for medicine than they are for advice. Advice is cheap, think they, and are not at all loth to occupy a half hour of the physician's time in describing their symptoms, asking useless questions, and walking off without ever thinking of pay;—it is *medicine* they expect to pay for, not advice. Hence are those physicians who keep their patients busy taking medicine, the most successful in making money.

An intelligent and conscientious physician never gives medicine unless it is necessary. More often will he direct some change in diet, clothing, bathing or exercise, or perhaps, may suggest some simple change in your daily habits, that to you, may seem unimportant, and for that reason may neglect his advice and seek some "medicine man" to provide treatment for you. People forget that the province of the physician is not only to prescribe medicine, but also to offer advice, and devise means for your guidance that will bring you safely through your illness and prevent a relapse. Would you not more willingly pay a physician who keeps you well, than one who doses you with physic every time you are taken ill, from lack of proper advise upon health matters? Of course you would. Therefore, when the physician is again summoned to your assistance, and when he gives you good and wholesome hygienic advice, and leaves you without medicine, when in his opinion none is necessary, pay him promptly for his visitation and his timely warning, the same as though the medicine had been left. Don't open your mouth to swallow a pill every time you meet your family physician.

---

GERMAN STUDENTS.—Large numbers of the students in German Universities have interrupted their studies, and have entered upon duty in the Ambulance Corps.

